# Early arrival did not ensure the early acquisition of intravenous thrombosis for acute ischemic stroke during the COVID‐19 pandemic

**DOI:** 10.1002/brb3.2977

**Published:** 2023-03-27

**Authors:** Huang Qiang, Sun Jin‐mei, Han Yan‐fei, Zhang Yong‐bo

**Affiliations:** ^1^ Department of Neurology Beijing Friendship Hospital, Capital Medical University Beijing People's Republic of China

**Keywords:** behavioral paradigms, cerebrovascular diseases, stress, stroke

## Abstract

**Background:**

Intravenous thrombolysis (IVT) in acute ischemic stroke (AIS) is a time‐dependent treatment with a narrow therapeutic time window, in which the time delay could result from the deadline effect.

**Methods:**

One hospital‐based cohort was recruited to detect the factors contributing to the deadline effect, where patients with the deadline effect were defined as those who were presented with the onset‐to‐door time (ODT) in the first 50%, while the door‐to‐needle time (DNT) was in the last quartile. DNT (in‐hospital delay) was further subdivided into several time intervals [door‐to‐examination time (DET), door‐to‐imaging time (DIT), door‐to‐laboratory time (DLT), and decision‐making time (DMT) of the patients or their proxies.

**Results:**

A total of 186 IVT cases were enrolled, of which 17.2% (32/186) suffered a delay of the deadline effect. The median age was 66 years, and 35.5% were female. Baseline characteristics were similar between the two groups (all *p* > .05). For the comparisons of the time intervals, DIT (26 versus 15 min, *p* = .001) was significantly longer in the group with deadline effect, while the differences of DET, DLT, DMT, and ONT did not reach statistical significance (all *p* > .05). Upon multivariable adjustment in the binary logistic regression model, longer DIT [odds ratio (OR), 1.076; 95% confidence interval (CI), 1.036–1.118; *p* < .001], and history of coronary heart disease (OR, 3.898; 95%CI, 1.415–10.735; *p =* .008) were independently associated with deadline effect in the binary logistic regression model, while admitted in the working day (OR, 0.674; 95%CI, 0.096–0.907; *p* = .033), and having medical insurance (OR, 0.350; 95% CI, 0.132–0.931; *p* = .035) were negatively associated with the deadline effect.

**Conclusions:**

A speed‐safety tradeoff phenomenon from the deadline effect was observed in 17.2% of IVT cases during the COVID‐19 pandemic, where longer DIT contributed a lot to this time delay. Patients without medical insurance, or admitted in official holidays were more likely to experience a delay of the deadline effect.

## Graphical abstract

1. Time delay due to deadline effect could have occurred in 17.2% (32/186) cases of the study, which made early arrival did not ensure early acquisition of intravenous thrombosis. 2. Behavioral and occupational factors in stroke physicians played an important role in time delay of deadline effect in intravenous thrombosis for acute stroke. 3. There was a speed‐safety tradeoff between integrating more information for potential improved safety and faster response time based on information already collected under urgent time pressure of decision making for intravenous thrombosis.

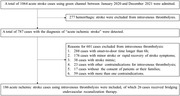

## 1 BACKGROUND

Acute ischemic stroke (AIS) is the leading cause of death in China in 2017 (Wu et al., 2019) and its prevalence continues to increase (Tu et al., 2022), in which intravenous thrombolysis (IVT) still serves as the mainstream drug therapy. According to several recent studies, the COVID‐19 pandemic was associated with a global decline in the volume of stroke hospitalizations, intravenous thrombolysis (IVT), and interfacility IVT transfers (Velilla‐Alonso et al., 2021; Xu et al., 2022). It was believed that the pandemic mainly affected postadmission treatment procedures, where prolonged time of the green channel for stroke during the COVID‐19 pandemic was the leading cause (Xu et al., 2022). Since the effectiveness and safety of IVT were high time‐dependent (Lees et al., 2010), where in‐hospital delay, also known as the door‐to‐needle time (DNT), consisted of a major component of the total time delay. Deadline effect, when physicians expect idle time following a task or just be occupied on other minor tasks (e.g., tasks for pandemic prevention), their work pace declines, and task completion time increases. It was estimated to have occurred in more than 20% of IVT patients after the extension of the time window from 3 to 4.5 h in European countries (Pitt et al., 2012). Deadline effect during the pandemic in such a time‐dependent urgency would have contributed a lot to the in‐hospital delay of IVT and counteracted the benefits of early arrival in saving the brain (known as “time is brain”; Saver, 2006). However, little was known about the deadline effect on Chinese doctors and there are significant variations in the Chinese health care system (Blumenthal & Hsiao, 2015), doctors’ situations (No Authors, 2014; Sun et al., 2017), and policies for the prevention of the pandemic to those in western countries. And workplace violence was more severer in Chinese hospitals than that western countries (Sun et al., 2017), which might bring into a different profile in the deadline effect of IVT. We are aiming at exploring the potential factors of deadline effect in IVT during the pandemic and illustrated how it worked through a hospital‐based cohort.

## 2 METHODS

### 2.1 Participants’ eligibility and enrollment

Consecutive AIS patients who were treated with IVT in our hospital between January 2020 and December 2021 were recruited retrospectively to detect factors that contributed to the deadline effect of IVT. A similar stroke pathway was fully available for 7 days a week and 24 h a day in our hospital with the help of doctor Huang and other colleagues (Huang et al., 2021), which was described in detail in previous studies (Huang et al., 2016; Huang et al., 2015). The inclusions and exclusions of IVT candidates followed the published Chinese guidelines (Chinese medical association neurological credit association, department of cerebrovascular diseases in Chinese medical association neurological credit Committee, 2015), namely, (1) age ≧18 years; (2) sudden and persistent neurological deficits [measured by the NIH stroke scale (NIHSS)]; (3) within 4.5 h of the stroke symptoms onset or the last normal time; (4) no hemorrhagic stroke in head CT and other contraindications listed in the guideline; (5) written consent achieved from the patients or their proxies.

### 2.2 Explanatory and outcome variables

Demographic data (including sex, age, and medical insurance status), baseline variables (severity of the stroke symptom, and blood pressure), medical history (hypertension, diabetes, dyslipidemia, coronary heart disease, atrial fibrillation, and prior stroke), smoking and drinking status, drug history, as well as additional factors likely to be associated with in‐hospital delay [such as lesion sites (classified as anterior and posterior circulation according to the results of later CT or MR imaging), admission date, and hour], were collected in forms of case reports. Admission date was divided into the working days and official holidays, while admission hours into working hours (from 8:00 to 18:00 between Monday and Friday) and nonworking hours (from 18:00 to 8:00 on the second day between Monday and Friday, and all day on weekend). The patients’ transportation methods were divided into using the emergency medical systems (EMS) and other methods.

Patients with the deadline effect were defined as those who presented with onset‐to‐door time (ODT) in the in first 50%, while the DNT was in the last quartile. DNT (in‐hospital delay) was further subdivided into several time intervals [door‐to‐examination time (DET), door‐to‐imaging time (DIT), door‐to‐laboratory time (DLT), and decision‐making time (DMT, defined as the time interval between the time obtaining the result of the last screening test and the IVT treatment time)].

### 2.3 Statistical analysis

Statistical calculations were using SPSS19.0 software, with two‐tailed *p* < .05 as statistically significant. The continuous variables with nonnormal distribution in Kolmogorov–Smirnov test were presented as median and interquartile range. Mann–Whitney *U* test and Pearson's chi‐square test were used for calculations of related variables. Pearson's correlation coefficient and significance test for the correlation coefficient were used to detect the association between related variables and deadline effect. Binary regression models were performed for multivariate analysis among related variables with a *p* value lower than .05 in Pearson's correlation, and covered potential predictors (age, gender, medical insurance status, transportation methods, admission date, and admission hour) to identify independent factors associated with the deadline effect.

## 3 RESULTS

### 3.1 Patients’ characteristics

**FIGURE 1:**
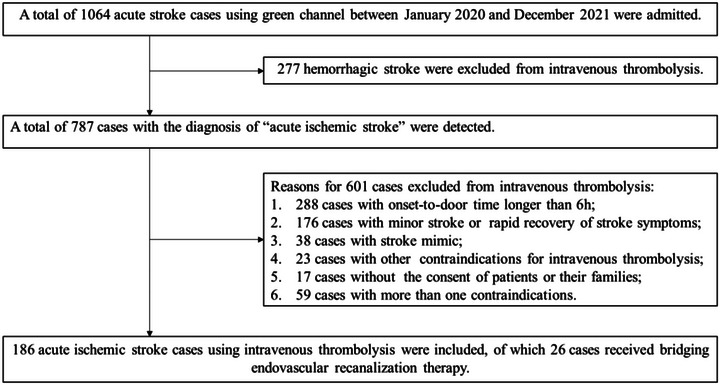
Research flow chart for this study.

As shown in the research flow chart in Figure 1, 186 IVT cases were recruited in the analysis, of which 32 (17.2%) suffered a delay that could be attributed to the deadline effect [which was measured as ODT in the first 50% (≦67 min), while the DNT in the last quartile (≧68 min)]. The median age was 66 (58, 75) years, and 35.5% were female in the total population. As shown in Table 1, baseline characteristics were similar between the two groups (all *p* > .05). And for the comparisons of the time intervals between two groups (Table 1), DIT (26 versus 15 min, *p* = .001) and DNT (98 versus 47 min, *p* < .001) were significantly longer, ODT (31 versus 81 min, *p* < .001) was significantly shorter in the group with deadline effect, while the difference of DET, DLT, DMT, and ONT did not reach statistical significance (all *p* > .05). Differences in clinical endpoints (modified Rankin Scale ≦2 at 3 months, symptomatic intracranial hemorrhage, recanalization, neurological improvement) also did not reach statistical significance (all *p* > .05).

**TABLE 1:** Baseline characteristics of cases included in this study.

	Total population (*n* = 186)	Patients with deadline effect (*n* = 32)	Patients without deadline effect (*n* = 154)	*p*
Age, years	66 (58, 75)	66 (61, 76)	66 (57, 75)	.744
Female	66 (35.5)	15 (46.9)	51 (33.1)	.139
Medical history				
Hypertension	123 (66.1)	24 (75.0)	99 (64.3)	.244
Diabetes	61 (32.8)	12 (37.5)	49 (31.8)	.533
Dyslipidemia	97 (52.2)	18 (56.3)	79(51.3)	.610
Coronary heart disease	49 (26.3)	14 (43.8)	35 (22.7)	.014
Atrial fibrillation	32 (17.2)	6 (18.8)	26 (16.9)	.799
Prior stroke	60 (32.3)	10 (31.3)	50 (32.5)	.893
Current smoking	92 (49.5)	8 (25.0)	84 (54.5)	.002
Heavy drinking	72 (38.7)	5 (15.6)	67 (43.5)	.003
NIHSS	8 (5, 14)	6 (4, 13)	9 (5, 14)	.441
Lesion in the anterior circulation	139 (74.7)	21 (65.6)	118 (76.6)	.193
Using EMS	77 (41.4)	14 (43.8)	63 (40.9)	.767
Working day	143 (76.9)	22 (68.8)	121 (78.6)	.230
Working hour	108 (58.1)	17 (53.1)	91 (59.1)	.534
Medical insurance	109 (58.6)	16 (50.0)	93 (60.4)	.278
Time intervals				
Door‐to‐examination time	3 (2, 4)	3 (1, 6)	3 (2, 4)	.381
Door‐to‐imaging time	17 (10, 22)	26 (12, 49)	15 (10, 20)	.001
Door‐to‐laboratory time	69 (55, 89)	66 (46, 93)	69 (56, 88)	.532
Decision‐making time	5 (4, 15)	5 (4, 28)	5 (4, 13)	.377
Onset‐to‐door time	67 (45, 121)	31 (21, 49)	81 (55, 130)	<.001
Door‐to‐needle time	53 (41, 68)	98 (80, 149)	47 (38, 57)	<.001
Onset‐to‐needle time	137 (104, 183)	138 (116, 178)	136 (97, 187)	.274
Clinical endpoints				
mRS ≦ 2 at 3 months	131 (70.4)	22 (68.3)	109 (70.8)	.819
Symptomatic intracranial hemorrhage	20 (10.8)	2 (6.3)	18 (11.7)	.555
Recanalization	109 (58.6)	19 (59.4)	90 (58.4)	.922
Neurological improvement^a^	121 (65.1)	18 (56.3)	103 (66.9)	.251

*Note*. Unless otherwise stated, continuous and categorical data are presented as median (IQR) and percentage (%), respectively, with *p* values calculated using Mann–Whitney *U* and χ^2^ tests, respectively.

^a^ Neurological improvement was defined as NIHSS equaled to 0 or improved by four points than baseline NIHSS at 24 h after intravenous thrombolysis.

NIHSS, National Institutes of Health Stroke Scale; EMS, emergency medical system; mRS, modified ranking scale.

After controlling factors of medical history (coronary heart disease, current smoking and heavy drinking), and DIT (all *p* > .05 in Pearson's correlation), as well as potential predictors (including age, gender, medical insurance status, transportation methods, admission date and admission hour), DIT (OR, 1.076; 95%CI, 1.036–1.118; *p* < .001) and history of coronary heart disease (OR, 3.898; 95%CI, 1.415–10.735; *p =* .008) were independently associated with deadline effect in the binary logistic regression model, while admitted in the working day (OR, 0.674; 95%CI, 0.096–0.907; *p* = .033) and having medical insurance [odds ratio (OR), 0.350; 95% confidence interval (CI), 0.132–0.931; *p* = .035] were negatively associated with deadline effect. The detailed result of the logistic regression model was shown in Table 2.

**TABLE 2:** Risk factors of deadline effect in all eligible patients.

Predictors of deadline effect	Odds ratio	95% Confidence interval	*p*
Age	0.981	0.943–1.020	.331
Sex	0.813	0.273–2.424	.710
Using EMS	0.767	0.283–2.079	.602
Coronary heart disease	3.898	1.415–10.735	.008
Current smoking	0.357	0.087–1.459	.151
Heavy drinking	0.674	0.148–3.068	.610
Working day	0.295	0.096–0.907	.033
Working hour	0.624	0.239–1.630	.336
Medical insurance	0.350	0.132–0.931	.035
Door‐to‐imaging time	1.076	1.036–1.118	<.001

## 4 DISCUSSION

There was a speed‐safety tradeoff phenomenon between integrating more information for potentially improved safety and faster response time based on information already collected under the urgent time pressure of decision‐making for IVT of acute stroke during the COVID‐19 pandemic. Even adopting a relatively conservative definition of the deadline effect, the time delay due to deadline effect could have occurred in 17.2% of IVT cases during the COVID‐19 pandemic. Longer DIT has independently associated with the deadline effect, in which time spent on obtaining head CT and additional chest CT images mainly corresponded to the time delay. And insufficiency of medical insurance or admission on official holidays might also have contributed to the deadline effect.

The time delay from deadline effect could have been partly due to a run on public medical resources during the COVID‐19 pandemic. That is because individuals run on medical resources for the pandemic, leading to inefficient usage and many unavoidable delays in acute stroke treatment, which could have included treatments for other time‐dependent conditions, such as myocardial infarction, pulmonary embolism, and so on. There was no significant association between deadline effect and mortality, which was greatly due to the small sample size. However, as showed in another nationwide study in China, during the COVID‐19 pandemic lockdown, patients hospitalized for stroke fell by 12.6%, and there were substantial increases in out‐of‐pocket rates (9.3%) and in‐hospital case fatality rates (18.0%) (Tu et al., 2023). Fair allocation of public medical resources in the time of COVID‐19 remains a huge challenge, since the COVID‐19 pandemic has led to an absolute scarcity of public medical resources, which could have affected all patients. Operationalizing the value of maximizing benefits in front‐line medical care workers, as well as policy makers, should be paramount during the pandemic.

Safety concerns rather than the efficacy of IVT were more focused on by Chinese doctors. On the one hand, although IVT was a proven efficient therapy with only a relatively increased risk of symptomatic intracranial hemorrhage, especially for those CHD patients who have usually taken antithrombotic agents, many emergency physicians (especially for nonneurologist physicians) were reluctant to take on the risk performing this therapy in clinical practice (Leira et al., 2007), and the decision‐making process for IVT could be much more time‐consuming. A questionnaire conducted on 719 neurologists from 66 Chinese hospitals showed that 32.9% of neurologists believed this treatment was unsafe, and 45.8% of neurologists felt unconfident about their ability to employ this treatment (Wang et al., 2018). Since head CT was the most important component for IVT decision‐making and could also serve good indicator (such as the hyperdense middle cerebral artery sign) for symptomatic intracranial hemorrhage (Jauch et al., 2013), a much longer time spent on obtaining head CT and sharing related information with the patients or their proxies showed in our study further revealed the safety concerns of doctors. On the other hand, faced with excessive occupational stress, additional medical resources taken up for the pandemic prevention and threat of workplace violence by Chinese doctors (Sun et al., 2017; Xu et al., 2022), as well as the burnout of stroke physicians who were usually involved in the round‐the‐clock task (working > 40 h per week) could have decreased the job performance of IVT (Wu et al., 2013). Patients admitted on official holidays were more likely to suffer the time delay of the deadline effect, which could have reflected the huge strain on human resources for the green channel of stroke during the COVID‐19 pandemic (Xu et al., 2022). Worth to note, limitations in physicians' knowledge regarding IVT (Ma et al., 2017) and the strict time pressure of urgent decision‐making also accounted for the lack of confidence in performing timely IVT. However, how it works in collecting more information for safety concerns at the cost of sacrificing time as the deadline approaches, and improving the quality of thrombolytic therapy during the pandemic needs further study.

The absence of medical insurance might have contributed to the time delay of the deadline effect. One reason for this phenomenon was that the personal financial burden of stroke treatment could have been a large expense for most people in China, especially for those who did not have on‐site medical insurance in Beijing (Yong et al., 2018). Since a real‐time settlement of off‐site medical insurance could not be achieved for most of the non‐Beijing patients at present time. Another reason was that, although the therapeutic time window of IVT has been extended to 4.5 h after stroke onset in the guidelines (Jauch et al., 2013), the administration of IV rt‐PA to patients presenting within the 3–4.5 h window after the stroke onset was still not adopted by the FDA (Daou et al., 2015) and CFDA. This off‐label thrombolysis was not covered by the present medical insurance policy in Beijing yet and needed a time‐consuming explanation in the decision‐making of IVT.

Given the large population suffering from AIS and the low rate of IVT for AIS (mainly due to time delay) in China (Xu et al., 2015), great progress seen in the TARGET‐Stroke program of the United States (Fonarow et al., 2014) was also ungently expected in China. Reducing the time delay of intravenous thrombosis played a key role, as the saying goes, time is brain, and 1.9 million neurons are dying per minute in AIS (Saver, 2006).

In this study, to the best of our knowledge, we first explored the time delay due to the deadline effect during the COVID‐19 pandemic, which may have indicated the importance of improving the organizational workflow and real‐time settlement of off‐site medical insurance to facilitate IVT in AIS. However, the main shortness of our study was the limited generalizability of the findings due to the small sample of a hospital‐based cohort and the retrospective study design, which thus needs further validation.

## 5 CONCLUSION

In a word, early arrival did not ensure the early acquisition of IVT during the COVID‐19 pandemic, where patients without medical insurance or admitted on official holidays were more likely to experience the time delay due to the deadline effect.

## CONFLICT OF INTEREST STATEMENT

The authors declare that they have no competing interests.

### INFORMED CONSENT

Informed consent was obtained from every participant.

### PEER REVIEW

The peer review history for this article is available at https://publons.com/publon/10.1002/brb3.2977.

## Data Availability

The data sets used during the current study would be available from the corresponding author upon reasonable request.
